# Predictive Modelling to Identify Near-Shore, Fine-Scale Seabird Distributions during the Breeding Season

**DOI:** 10.1371/journal.pone.0150592

**Published:** 2016-03-31

**Authors:** Victoria C. Warwick-Evans, Philip W. Atkinson, Leonie A. Robinson, Jonathan A. Green

**Affiliations:** 1 School of Environmental Sciences, University of Liverpool, Liverpool, United Kingdom; 2 British Trust for Ornithology, The Nunnery, Thetford, Norfolk, United Kingdom; Centre for Ecological and Evolutionary Studies, NETHERLANDS

## Abstract

During the breeding season seabirds are constrained to coastal areas and are restricted in their movements, spending much of their time in near-shore waters either loafing or foraging. However, in using these areas they may be threatened by anthropogenic activities such as fishing, watersports and coastal developments including marine renewable energy installations. Although many studies describe large scale interactions between seabirds and the environment, the drivers behind near-shore, fine-scale distributions are not well understood. For example, Alderney is an important breeding ground for many species of seabird and has a diversity of human uses of the marine environment, thus providing an ideal location to investigate the near-shore fine-scale interactions between seabirds and the environment. We used vantage point observations of seabird distribution, collected during the 2013 breeding season in order to identify and quantify some of the environmental variables affecting the near-shore, fine-scale distribution of seabirds in Alderney’s coastal waters. We validate the models with observation data collected in 2014 and show that water depth, distance to the intertidal zone, and distance to the nearest seabird nest are key predictors in the distribution of Alderney’s seabirds. AUC values for each species suggest that these models perform well, although the model for shags performed better than those for auks and gulls. While further unexplained underlying localised variation in the environmental conditions will undoubtedly effect the fine-scale distribution of seabirds in near-shore waters we demonstrate the potential of this approach in marine planning and decision making.

## Introduction

Seabirds are primarily suited to life at sea, however during the breeding season they are constrained to coastal areas, often breeding in large colonies, and rafting and foraging in the coastal waters adjacent to breeding sites [1]. At the same time, the potential for negative interactions between humans and seabirds is particularly acute in coastal areas, since seabirds have to use these areas and human activities are concentrated in near-shore locations [2]. Understanding how the vulnerability of seabirds varies for different types of anthropogenic disturbance, requires information on how likely they are to interact with an activity (exposure) and the severity of effects where interaction occurs (sensitivity) [3,4]. Severity of effects is well documented for some interactions [5,6] and less well understood for others [7,8]. Likely exposure to activities requires an understanding of the factors driving distributions of seabirds in space and time.

The factors associated with seabird distributions include, but are not limited to, environmental factors such as bathymetry [9,10], distance to land and nest site [9,11], substrate type [12], chlorophyll levels [13], sea surface temperature [14] and oceanographic processes [15,16]. Many of these and their interactions may be proxies for the underlying factors influencing seabird distribution which are primarily prey availability and energetic constraints. In addition, ecological interactions, such as local enhancement [17] or competition [18] may be important. Furthermore, prey availability and important at-sea areas, may vary temporally [9,19,20], or be dependent on weather conditions [9,21]. Although widely studied, most research into the factors driving seabird distribution is conducted at moderate to large spatial scales, and very fine-scale distributions are rarely considered. Yet it is the factors affecting near-shore, fine scale distribution that are most pertinent when considering marine spatial planning issues, such as the licensing of new human activities in coastal areas.

Methods for studying seabird distributions include: large-scale ship-based or aircraft surveys of all species in a pre-defined area [22]; and novel tracking technologies which provide very fine-scale location and behavioural information for a sample of individuals from a known colony [23]. These methods have improved our understanding of seabird habitat use and at-sea distributions, as the interactions between the physical and biological environments and how they influence seabird distributions are explored. However while seabird tracking studies, in particular, have improved our understanding of seabird ecology, they are not always feasible, as recommended guidelines on the load of biologging devices [24] preclude small birds from carrying some devices, and some species and populations are not amenable to tracking [25]. Additionally often only subsets of the population are tracked which could induce bias [26], and tracking data is colony specific rather than site specific i.e. birds from that colony may not enter the area of interest. Additionally, large scale aircraft or ship-based surveys are expensive, and can be problematic in shallow and topographically complex habitats. Shore-based surveys overcome these aforementioned issues, and for near-shore fine-scale studies, it should be possible to use vantage point observations. Vantage point observations have been used to gain presence-absence data of seabirds in areas proposed for the development of offshore renewable energy devices, however these distributions have not been related to the underlying environmental variables, presumably because most surveys in coastal environments are driven by environmental impact assessments which only focus on quantifying numbers of birds in the site, rather than their habitat use [22]. This approach has been used successfully to investigate distributions of marine mammals in this context [27]. Furthermore these observations could allow behaviours such as flocking, foraging and fine-scale interactions between seabirds and the environment to be monitored.

For example, Alderney and its surrounding islands in the English Channel are important breeding grounds for many species of seabird (Table 1) and also one of the best locations world-wide in its potential for harvesting tidal stream energy on a large-scale [28]. With consistently high current speeds coupled with depths of 25 m—45 m it is an ideal environment for the operation of tidal turbines [29] and Alderney Renewable Energy (ARE) has been granted the license to install a tidal stream array to exploit this resource. In addition Alderney is a popular destination for recreational boating, and proposals for a marina are being discussed [30], thus there is the potential for high levels of exposure to anthropogenic disturbance. In order to understand how developments such as these are likely to affect seabird populations such as those in Alderney, it is necessary to understand the drivers behind their fine-scale distribution in near-shore waters [31]. We use vantage point observations of the distributions of seabirds around Alderney, collected during the 2013 breeding season, to identify and quantify some of the environmental variables affecting their fine-scale distributions. We validate the model using observation data collected in 2014. In doing so we have developed a simple yet powerful observation and modelling approach that could be used in other locations in order to examine potential impacts of anthropogenic disturbance operating in the near-shore environment.

**Table 1:** Seabirds breeding on Alderney and its surrounding islands.

Species	Number of breeding pairs	Importance
Atlantic puffin *Fratercula arctica*	143	Largest in English Channel
Storm petrel *Hydrobates pelagicus*	2800^a^	Regionally important
Lesser black-backed gulls *Larus fuscus*	1392	Nationally important
European shag *Phalacrocorax aristotelis*	167	Nationally important
Common guillemot *Uria aalge*	120^b^	
Razorbill *Alca torda*	90^b^	
Northern gannet *Morus bassanus*	7885	Internationally important

^a^. Number of individuals (2008).

^b^. Approximation (Pers Comms, Alderney Wildlife Trust).

Data extracted from [32].

## Materials and Methods

### Study area

**Fig 1:**
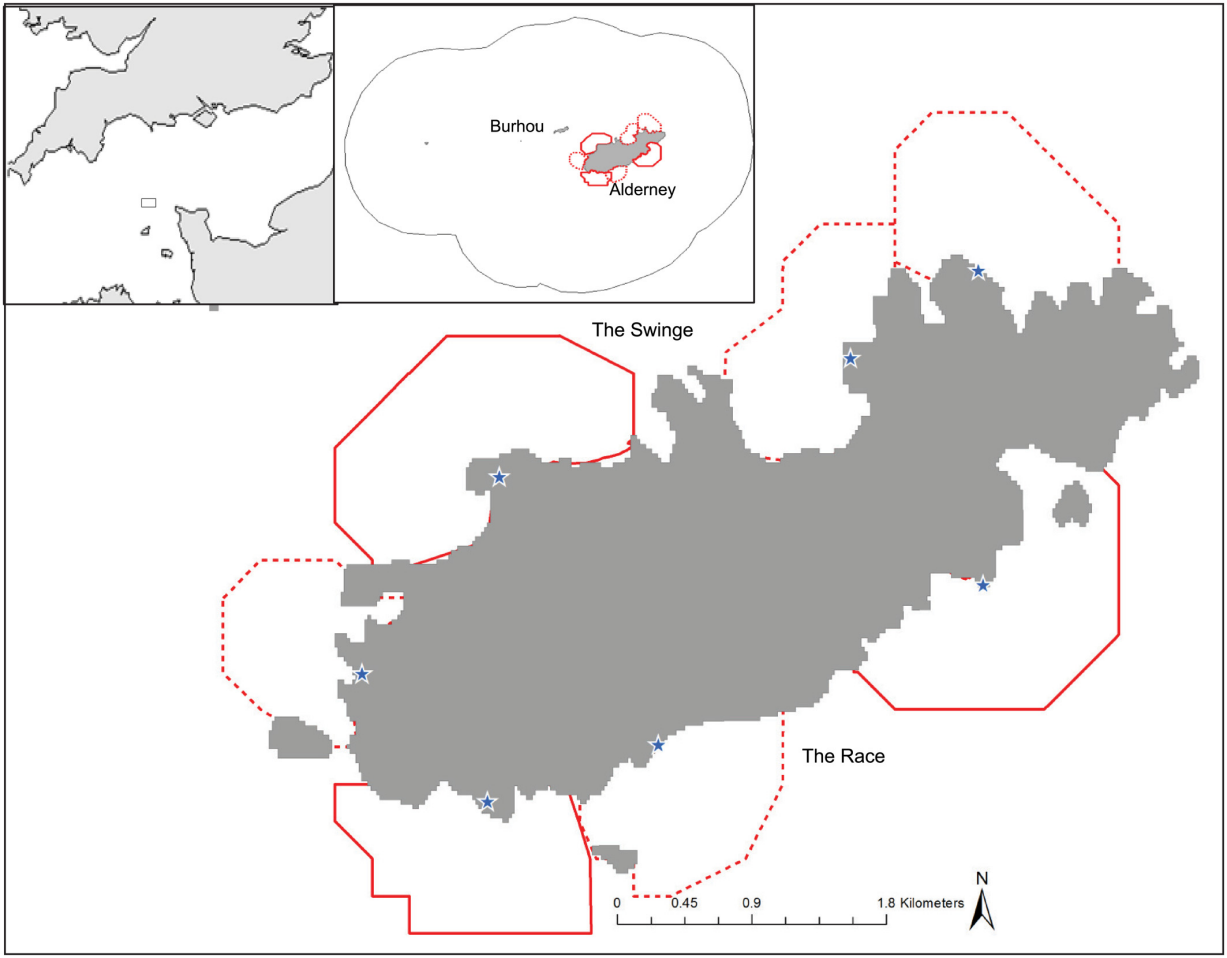
Alderney and the island of Burhou (inset). The locations of vantage point observations conducted in 2013 (solid red lines) and 2014 (dotted and solid red lines) are marked on the map.

Alderney, Channel Islands (49 42' 50" N, 2 12' 18" E) is renowned for its fast flowing tidal stream which divides around the island creating The Race to the south and The Swinge to the north (Fig 1). Currents in these waters can exceed speeds of 2.5 ms^-1^ [28]. In addition the tidal range is large, so there are large intertidal zones and many of the rocks and islets which are prevalent in Alderney’s near-shore waters only protrude from the water at low tide.

We attempted to quantify this spatial variability in the near-shore environment by defining a number of environmental variables on a 250 m x 250 m grid which included all areas up to 1 km from the coast of Alderney using ArcGIS 10.2 (ArcGIS Desktop: Release 10. Redlands, CA: Environmental Systems Research Institute, S1 Fig). These variables were: euclidean distances to intertidal areas (low water mark, including offshore intertidal rocks), to land (high water mark), to nearest nest and to groups of 5, 10 and 20+ nests, mean depth (extracted from an admiralty chart, range from 0 (in the intertidal zone) to 30 m), and the substrate type (coarse sediment or circalitoral rock [33]). Unfortunately no data was available on the fine-scale tidal flow speeds in Alderneys near- shore waters, and therefore we were unable to include this in the model. All maps were downloaded from the GADM database of global administrative areas [34].

### Bird Observations

**Fig 2:**
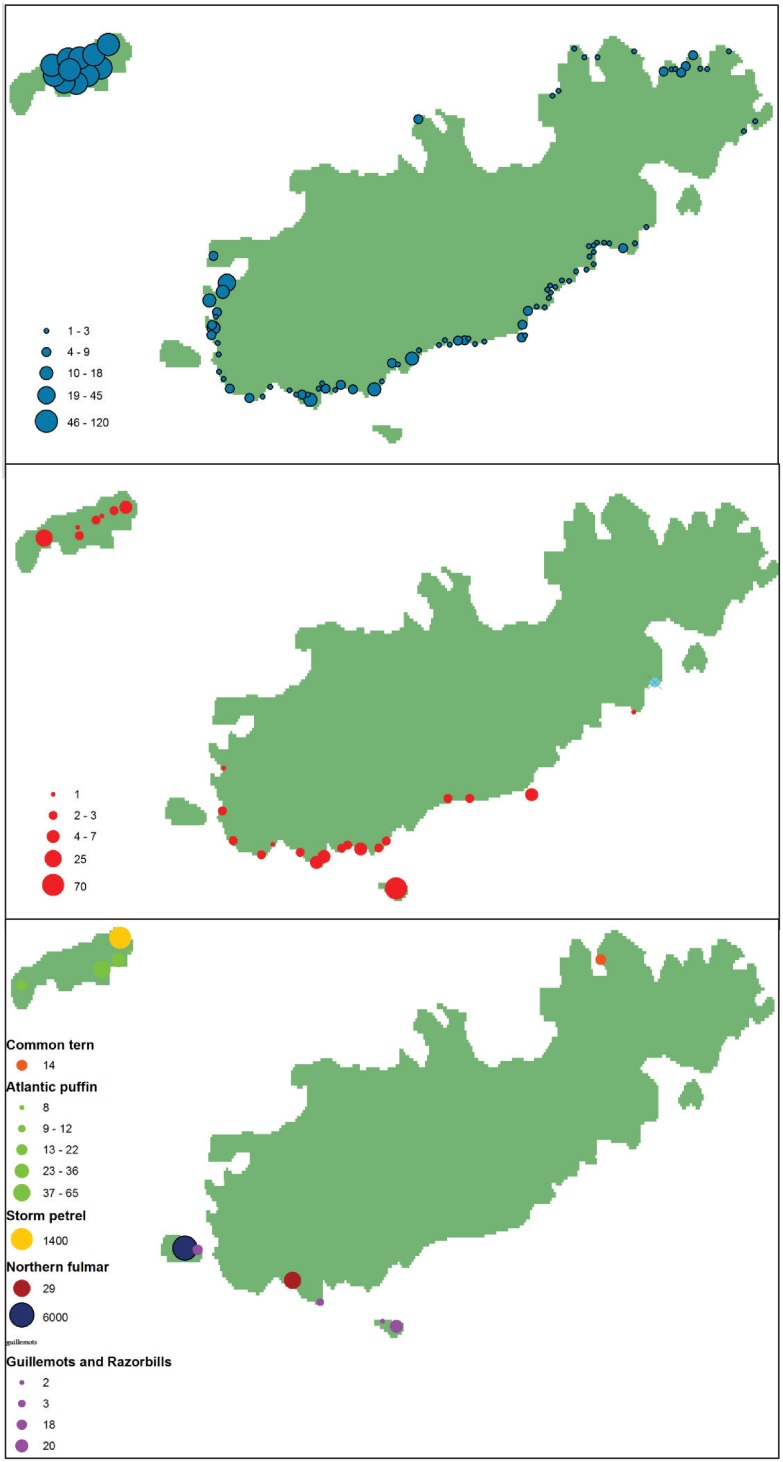
Distribution of seabird breeding sites on Alderney. (a) large gulls, (b) shags, (c) other seabirds nesting on Alderney in 2013.

The number of nests and their locations on Alderney and Burhou was mapped for each species from boat and foot-based surveys. Shags, gulls, large auks and gannets nest on the south cliffs of Alderney and the islets to the south and west while the island of Burhou, approximately 2.5 km to the north west, hosts more shags, gulls, puffins and storm petrels (Fig 2). Land based vantage point observations of birds at sea were carried out on Alderney, during the seabird breeding season (April—July) in 2013 and 2014. Fieldwork on Burhou (i.e nest counts) was carried out as part of the RAMSAR management plan which is authorised by the states of Alderney and maintained by the Alderney Wildlife Trust. No permission was necessary for fieldwork on Alderney as this was all carried out on public land, and nests were not approached. The fieldwork did not require handling any animals therefore no permissions from animal ethics committees were required.

In 2013 three vantage points were selected, one site overlooking The Swinge, and two overlooking The Race (Fig 1). In 2014 a further four sites were added. Each vantage point was over 30 m above sea level in order to obtain a good view over the observation area, which extended up to 1km from the vantage point in all seaward directions. On each visit 4 seabird distribution scans were conducted in order to maximise the likelihood of detecting diving birds, each taking approximately 15 minutes. For each scan, binoculars (7x50) were used to scan the observation area and birds on the water were identified, a bearing was taken, and a rangefinder was used to estimate the distance to the bird at the location the bird was first sighted. Identification of the birds to species level was not consistently possible, therefore birds were classified into broader groups of large gulls (comprising great black-backed *Larus marinus*, lesser black-backed and herring gull *Larus argentatus*), large auks (comprising common guillemots and razorbills), and European shags. The occasional Atlantic puffin was also observed, but the sample size (4 observations) was insufficient for these to be included in the analysis. Northern gannets were also excluded from the analysis as there were very few sightings of foraging birds (on only one occasion there were plunge diving gannets in the observation area), and many loafing adjacent to the large colony at Les Etacs, with few sightings elsewhere around the island. No other species of seabird were observed in the observation areas.

Although behaviour was not recorded, all species were observed loafing on the water and foraging, either by diving (large auks and shags), or dipping from the surface (large gulls), in all locations where they were observed. In 2013 observations were carried out at all sites up to 6 days a week for 4 months (April—July) resulting in a total of 65 days of data for each site. In 2014 each of the seven vantage points was visited weekly, resulting in a total of 16 days of observation data for each observation area (S1 Table). Observations were not carried out in bad visibility (< 2000 m) or in sea state greater than 4 (~ 95% were in sea state 1–3), and this, combined with the height of the vantage point and the relatively close distance to the edge of each site, means we were confident that all birds in the observation area were seen. There was not sufficient time to incorporate the potential effects of the state of tide with either time of day or day of year with a suitable number of repeats. Thus, the vantage points were visited at the same time every morning (08:00–12:00), to ensure that the time of day was consistent, but all states of tide were incorporated in the observations. The state of tide (ebb, flood or slack) and the time since high tide (expressed as both a continuous variable, and categorically grouped into two hour blocks) at each site was calculated for every visit.

### Analysis

Each group (shags, large gulls and large auks) was analysed separately owing to differences in the ecology and foraging behaviour between the three groups. The latitude and longitude for each bird sighting was calculated from the distance and bearing from the vantage point using the geosphere package in R [35]. For each visit a single scan containing the maximum number of sightings was selected, with the aim of including all birds that were in the observation area, including those that were diving during some scans, whilst avoiding double counting. The bird locations were added to the grid of environmental data and mean values of all explanatory variables were calculated for each grid cell: These values, as well as the presence or absence of seabirds in each grid cell on each visit, were exported from GIS and analysed using R (version 3.0.2, R Development Core Team 2013).

#### Model

For each group a generalised linear mixed model with a binomial error structure was created using the *glmer* function in package *lme4* [36]. Presence—Absence models were used due to their robustness in situations such as ours with zero-inflated datasets, additionally our aim was to keep the analysis simple, thus more complex methods to calculate spatial distributions were not used in this instance. Models were constructed to calculate which of the environmental variables affected the probability of finding at least one bird of that group in a given grid cell using data collected in 2013. In each case the explanatory variables in the starting model were distance to land, distance to the intertidal zone, distance to the nearest conspecific nest, distances to nearest groups of conspecific nests (5–9, 10–19, 20+), depth, substrate type, and all measures of tidal state for each observation. We did not have sufficient data to include either time of day or day of year as a variable, thus the whole breeding season was treated as a single time period. Each grid cell was included as a random effect in order to take account of the repeated observations in each cell. Variables were scaled and centred in order to improve interpretation [37]. The model with the lowest Akaike Information Criterion (AIC) score of all of the possible combinations of explanatory variables was determined using the *dredge* function in the *MuMIn* package [38]. Likelihood ratio tests were used to obtain the significance for each explanatory variable in the final model. The model was then used to predict the probabilities of observing a bird of that group in each cell within the seven observation sites surveyed in 2014. There was some non-independence between the covariates, however a correlation coefficient of 0.65 between our most correlated variables; depth and distance to the intertidal zone, is below the accepted threshold of 0.7 for regression models [39]. Plots of the shape of these correlations were curved, suggesting that the variables were not simply covarying, thus justifying their retention [39].

The model was validated against the proportion of times a bird of that group was observed in each grid cell over 16 visits in 2014.

**Fig 3:**
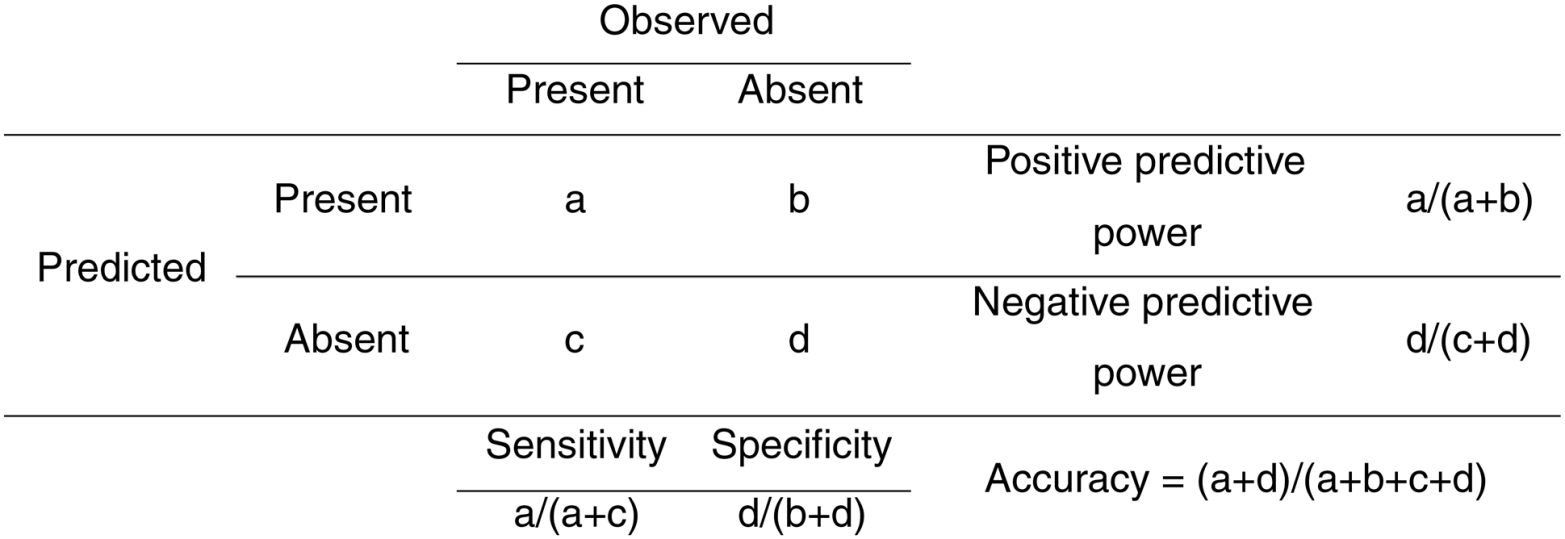
A confusion matrix. This describes how accuracy, sensitivity, specificity, negative predictive power and positive predictive power are calculated.

A Receiver Operating Characteristic (ROC) curve was created in R package pROC [40] in order to test the model for errors of omission (falsely predicted negative values) and commission (falsely predicted positive values) [41]. The ROC curve is a plot of true positive values (sensitivity) against 1- the false positive values (specificity), for all available thresholds of movement between classes (i.e the point at which absent becomes present). The “best” threshold is considered to be that where the difference between sensitivity and specificity is least [41]. The Area Under the Curve (AUC) was calculated to test the overall performance of the model [42]. AUC may range from 0.5 to 1, where a value of 0.5 is no better than random, and a value of 1 would be a perfect model [41]. Accepted thresholds for model performance are; low accuracy (0.5–0.7), useful applications (0.7–0.9) and high accuracy >0.9 [43]. In addition the positive predictive power (ppp), negative predictive power (npp), sensitivity and specificity were calculated (Fig 3).

The validated model was then used to predict the distribution of seabirds in the coastal waters surrounding Alderney. Predictions of the probability of finding a bird in a given cell were made up to 1 km from the coast of these islands. The environmental conditions of these waters were all within the same ranges as those in the original observation areas.

## Results

Of the 117 grid cells surveyed in 2013 there were 83, 49 and 65 cells with at least one observation of a shag, auk and gull respectively. Of the 217 grid cells surveyed in 2014 there were 78, 48 and 78 cells with at least one observation of a shag, auk and gull respectively. This difference in the number of grid cells used was due to the birds tending to use the same grid cells in both years. Few seabirds were found in the new observation areas.

**Fig 4:**
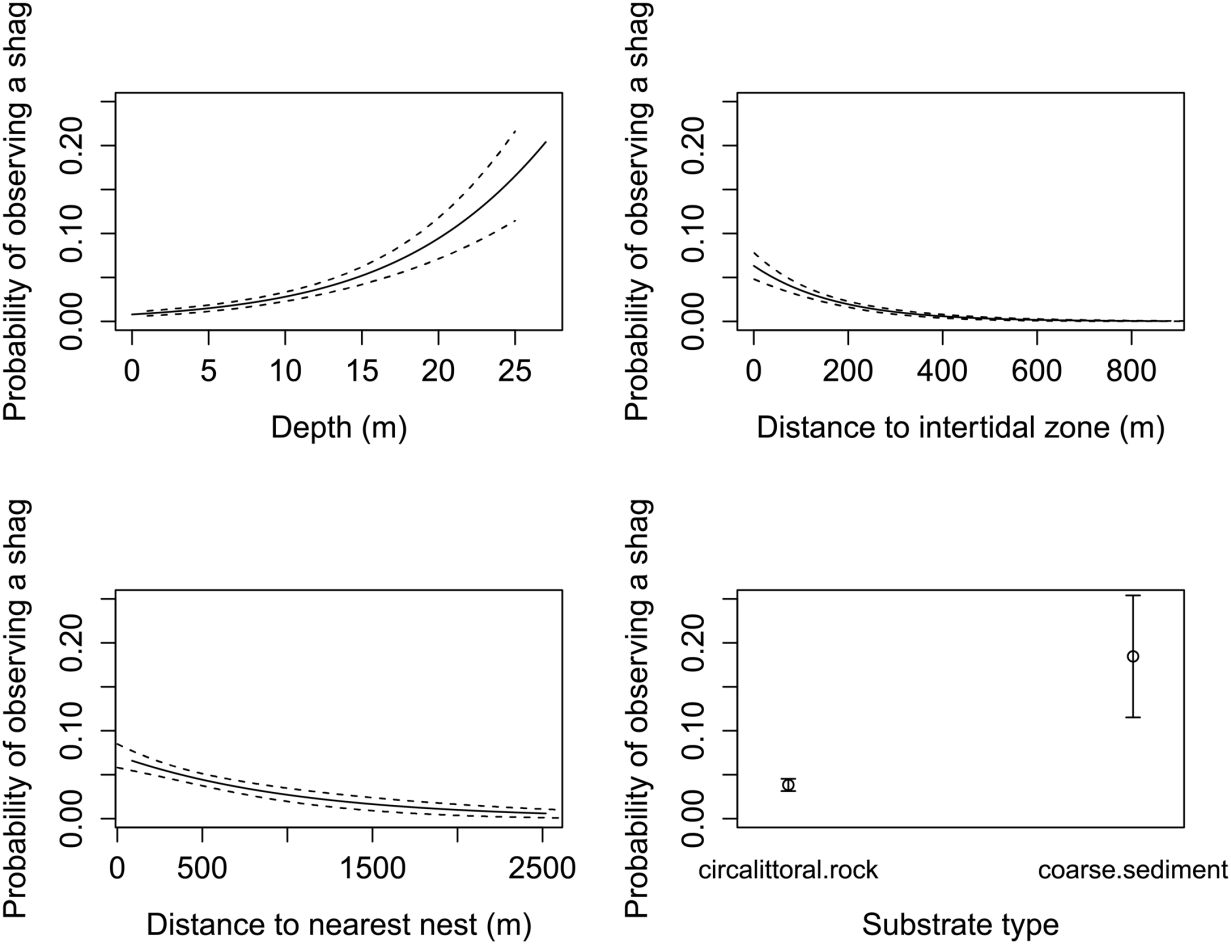
Explanatory variables to describe the near-shore distribution of European shags. The probability (and standard error) of observing a European shag as a function of a) depth, b) distance to intertidal zone, c) distance to nearest seabird nest and d) substrate type considered independently, and not accounting for the combined effects of these environmental variables, and are adjusted for the median value for the other numerical predictors in the model, and for the reference level for factors. Based on vantage point observations of the distribution of shags in Alderneys coastal waters over 65 days during the 2013 breeding season.

**Fig 5:**
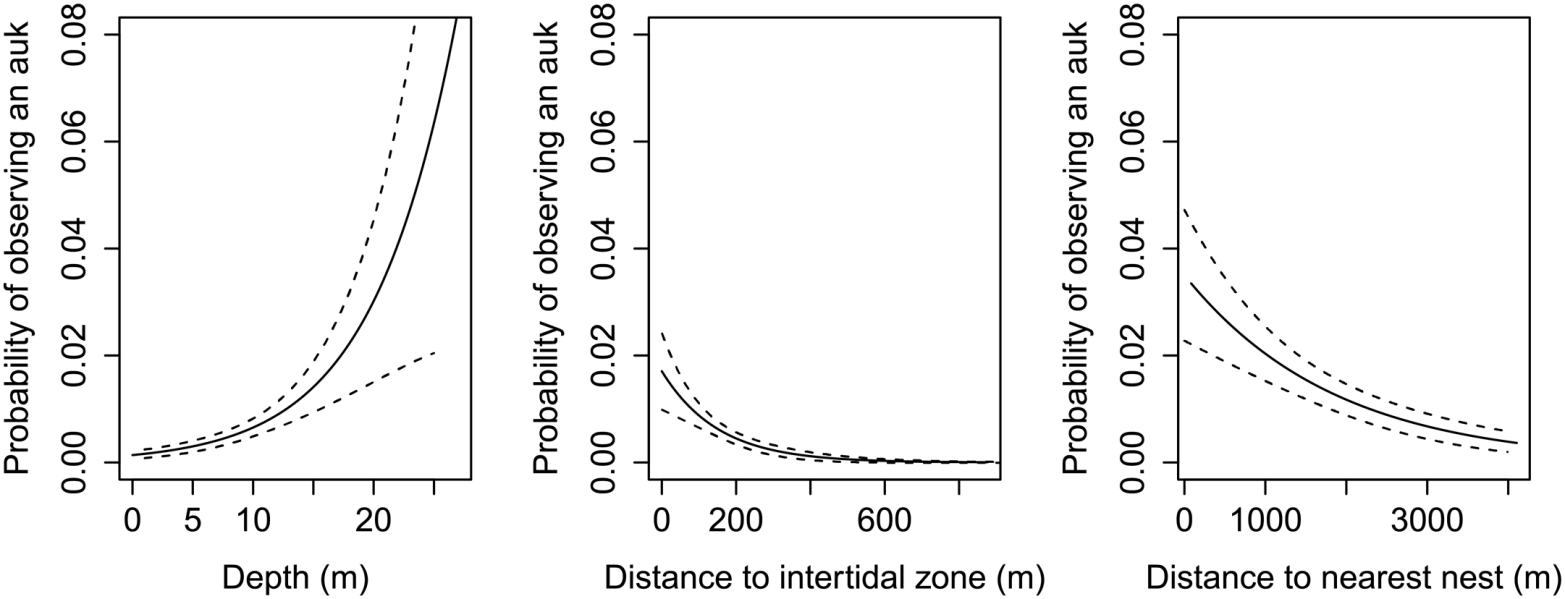
Explanatory variables to describe the near-shore distribution of large auks. The probability (and standard error) of observing an auk as a function of a) depth, b) distance to intertidal, c) distance to nest considered independently, and not accounting for the combined effects of these environmental variables, and are adjusted for the median value for the other numerical predictors in the model. Based on vantage point observations of the distribution of shags in Alderneys coastal waters over 65 days during the 2013 breeding season.

**Fig 6:**
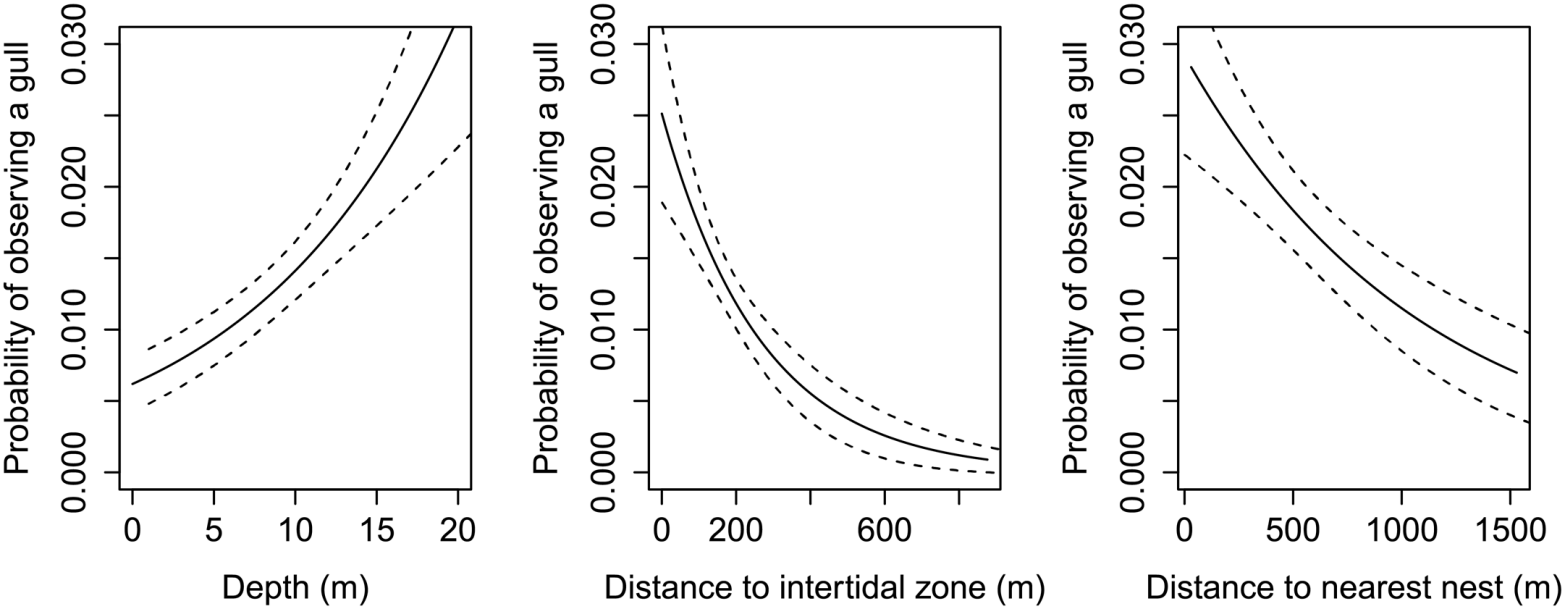
Explanatory variables to describe the nearshore distribution of large gulls. The probability (and standard error) of observing a large gull as a function of a) depth, b) distance to intertidal, c) distance to nest considered independently, and not accounting for the combined effects of these environmental variables, and are adjusted for the median value for the other numerical predictors in the model. Based on vantage point observations of the distribution of gulls in Alderneys coastal waters over 65 days during the 2013 breeding season.

The near-shore, fine-scale distribution of all three groups of seabirds which make up the majority of the birds observed around Alderney can be partially explained by distance to the nearest seabird nest, distance to the intertidal zone and depth (Table 2). Substrate type was also important for shags. The probability of observing a shag (Fig 4), auk (Fig 5) or gull (Fig 6) was higher in areas closer to the nest and the intertidal zone, and in deeper water (Table 2). The probability of observing a shag was higher over coarse sediment substrates (Fig 4).

**Table 2:** Environmental variables to describe the distribution of Alderneys seabirds.

Group	Variable	Estimate	Std Error	p-value
European shags	Distance to the intertidal zone	-1.2	0.23	<0.001
	Depth	1.11	0.21	<0.001
	Distance to nearest nest	-0.72	0.27	0.007
	Substrate (coarse sediment)	0.83	0.27	0.003
Large auks	Distance to the intertidal zone	-1.29	0.45	0.002
	Depth	1.35	0.39	<0.001
	Distance to nearest nest	-0.67	0.21	0.001
Large gulls	Distance to the intertidal zone	-0.71	0.26	<0.001
	Depth	0.63	0.20	0.001
	Distance to nearest nest	-0.33	0.14	0.02

Legend: Significant environmental variables scaled and centred (likelihood ratio p-values) in the models to predict the distribution of seabirds in Alderneys coastal waters.

AUC values of 0.66–0.78 calculated from the ROC curve suggest that overall the performance of all the models is fairly good. However, correct classifications of 57–77% suggest that the model for shags is good and superior to that for auks and for gulls (Table 3). Sensitivity (the correctly predicted presence observations) and specificity (the correctly predicted absence observations) of the models were also good (0.63–0.82 and 0.56–0.78 respectively, Table 2). In addition the negative predictive power (i.e. the proportion of predicted absences which are also observed absences) was extremely high (97–99%). However the positive predictive power (i.e. the proportion of predicted presences which were observed presences) was low (6–13%, Table 3).

**Table 3:** Model scores from a ROC curve.

Group	Threshold	Correct classification (%)	PositivePredictivePower (%)	NegativePredictivePower (%)	Sensitivity	Specificity	Area under curve
European shags	0.09	77	13	98	0.63	0.78	0.73
Large auks	0.006	61	6	99	0.82	0.61	0.78
Large gulls	0.014	57	7	97	0.68	0.56	0.66

Legend: Based on a presence-absence model using environmental variables to predict the fine-scale distribution of seabirds in Alderney’s coastal waters.

**Fig 7:**
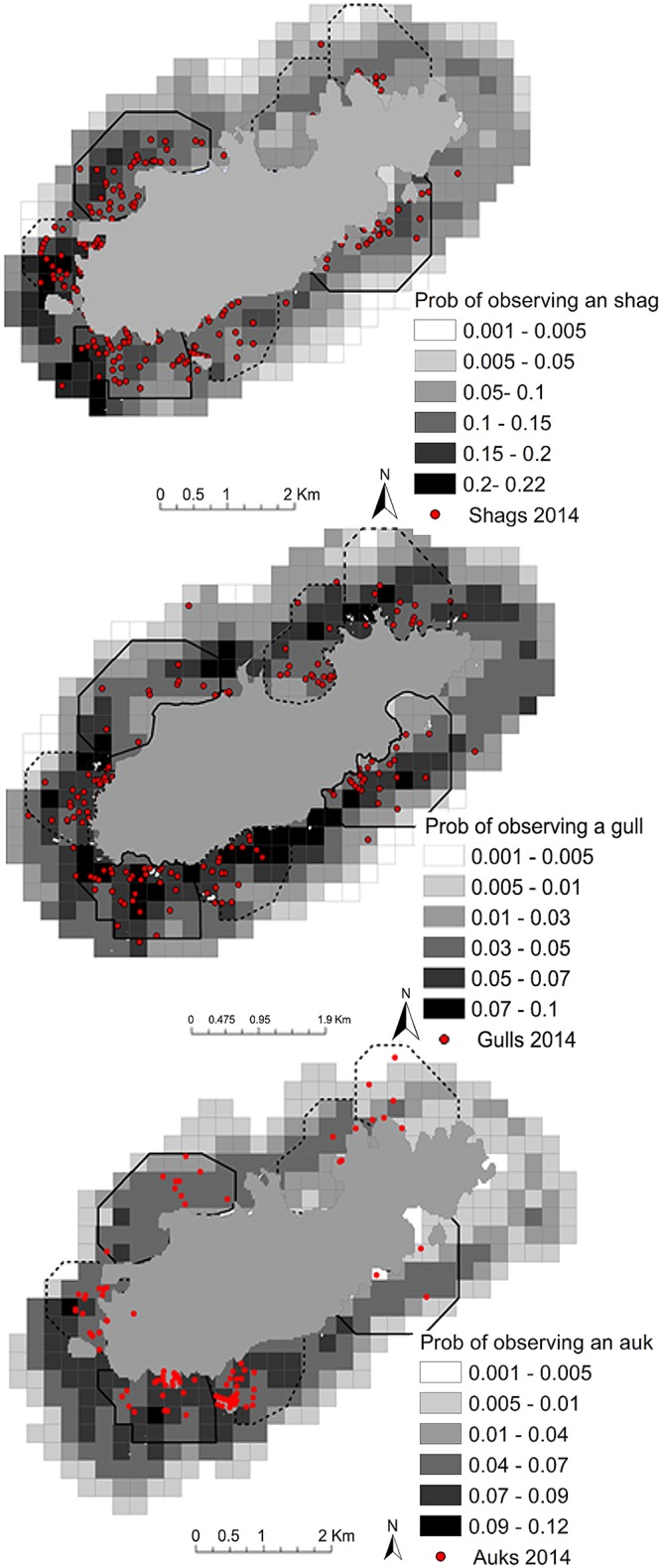
Predicted and observed distributions of Alderneys seabirds. The probability of observing a) a shag, b) a gull, c) an auk within 1 km from the coastline of Alderney. Sites surveyed for birds in 2013 (continuous black line) and 2014 (dotted and continuous black line) are marked. Predictions were made based on a presence-absence model using observations made in 2013 and verified with observations made in 2014.

In 2014 birds from all three groups were observed most often in waters off the south west coast of Alderney, nearest the majority of nest sites and in line with predictions of suitable habitats by the models. Shags and gulls (Fig 7a and 7b) were observed along the south coast and tended to remain within 500 m from the coast. Auks were rarely observed off the south-east coast and tended to remain towards the west of the island (Fig 7c).

## Discussion

In order to understand the potential for any negative impacts from human activities a comprehensive knowledge of the distribution of seabirds with the potential to be affected at a relevant spatial scale is vital. Presence-absence models show that the near-shore, fine-scale distribution of seabirds in Alderney’s coastal waters can partially be explained by distance to the intertidal zone, distance to the nearest seabird nest, depth and substrate type. Overall classification rates and AUC values indicate that the binomial models perform reasonably well for shags and auks, and less well for gulls. In particular the models were highly accurate at predicting where the birds were unlikely to be found, but tended to over-estimate the presence of birds, suggesting that factors other than those considered in our study are important in determining habitat use and at-sea distribution. Studies of seabird biology tend to focus on foraging trips and the literature on seabird habitat use is dominated by telemetry studies of presumed foraging birds at sea. However, a bird observed at sea is not necessarily foraging; seabirds also rest and raft at sea [1,44], and this aspect of their behaviour is understudied. Some of our birds were observed foraging, but our study shows that whether foraging or not, at a fine-scale, birds do not use the near-shore environment randomly and have clear preferences for some areas. Thus, the factors underlying their distribution should be considered with respect to decision making for coastal developments. The following discussion will focus on the important variables driving the near-shore fine-scale distribution of seabirds as identified in the model.

### Environmental variables

At-sea distributions of foraging seabirds are considered to be driven primarily by prey distribution, but restricted by behavioural, morphological and energetic constraints of the bird [20]. Many previous studies describe the influence of various environmental and oceanographic variables on the at-sea distribution of seabirds [9,15], however most studies are conducted at a relatively large spatial scale in comparison to this one. Seabirds appear to make hierarchical decisions, firstly to identify large-scale suitable foraging areas, and then, nested within these areas, to utilise fine-scale habitat features which aggregate prey [45]. Therefore, environmental and oceanographic variables may have different relative importance at different spatial scales [20]. In addition seabirds display temporal variation in their distributions, most prominently between the breeding and non-breeding seasons, and it is important to understand their distributions during both of these periods. Tidal state, which can be linked to current speed [46] was found not to be important in our model. This contrasts with other studies of seabirds in areas of high tidal flow. Since most of our birds were close to the intertidal zone they may have been isolated from these current effects found in the water further offshore. As noted earlier, unfortunately we were unable to obtain fine-scale current data for this area. We establish that depth, distance to the nearest seabird nest, distance to the intertidal zone and substrate type are important factors influencing the distribution of Alderney’s seabirds.

#### Distance to the intertidal zone

A higher probability of observing all three bird groups closer to the intertidal zone may be explained by an increase in prey availability in these locations. Many intertidal and subtidal rock formations surround Alderney’s coastline and birds were frequently observed in these areas. This type of feature is likely to enhance the occurrence of small scale eddies and shear lines which can aggregate prey in predictable locations [47]. These oceanographic processes are important for foraging seabirds, at both large [15,19] and small [45,48] spatial scales. Sandeels are the primary prey type for auks and shags during the breeding season and are likely to aggregate in these areas. Furthermore, previous studies have revealed that they are able to supplement their diet with crustaceans [49–51], which are common in this habitat type. In addition, gulls frequently forage in the low intertidal and shallow sub-tidal zones [52] on benthic crustacean and small fish [53].

#### Distance to nest

The energetic cost of foraging increases with the distance travelled to foraging locations, unless there is variability in the cost of foraging, or the energy gained from prey. Thus it is logical for birds to exploit available prey patches in close proximity to the nest. Previous studies demonstrate how distance from the colony is an important factor in the at-sea distributions of guillemots [54] and shags [55]. In addition, although we know that time spent in an area can be used as a proxy for foraging behaviour [56], seabirds also spend time rafting near to their colonies for purposes such as information exchange [44]. Although foraging behaviour was observed in all groups, the frequency of this behaviour was not recorded, and these areas may be used primarily for loafing rather than foraging. However, as the focus of the study is to understand seabird distribution and not specifically active foraging sites, all locations are relevant.

#### Depth

Many species of seabird forage in water of a preferred depth [55,57], presumably due to increased prey availability in these locations. It has been suggested that when considering the fine-scale distribution of top-predators, processes which increase prey aggregation are more important than the oceanographic processes driving primary production [45]. In the absence of detailed data on preferential prey and the distribution of prey, we can only assume that birds select these greater depths based on increased prey availability. In addition, this deeper water may contain topographical features such as sea banks and tidal forcing associated with these features may cause the aggregation of zooplankton [58] leading to fish aggregation and therefore superior foraging locations.

#### Substrate type

Shags were encountered more often over areas of coarse sediment than over rocky substrates. Although shags are able to forage for sandeels in both the pelagic and benthic zones [59], their diving strategy is considered to be primarily benthic [12], consuming bottom living fish and probing the sand for buried sandeels [12]. Consequently, this explains the increased probability of observing a shag in areas of coarse sediment. Although guillemots and razorbills also primarily forage for sandeels during the breeding season [60], they are pelagic feeders, and do not exploit sand dwelling fish, hence substrate type is likely to be less important in the distribution of auks. Gulls do not dive at all, and forage by scooping fish from surface waters. Thus, substrate type is not an important variable driving their at-sea distribution.

### Model performance

The models predicting the distribution of shags and auks perform reasonably accurately when evaluated using the AUC values and the percentage of correct classifications. AUC values are frequently used to assess the performance of presence-absence models [61,62]. However these may not accurately represent key aspects of model performance [63,64] such as errors of commission and omission. Deconstructing models to evaluate separate measures of prediction success, based on errors of commission or omission may be more suitable [41,64]. Sensitivity and specificity measure the proportion of observed presences and absences which are correctly predicted, respectively. Positive predictive power (ppp) and negative predictive power (npp) measure the proportion of predicted presences and absences which were also observed i.e the proportion of true presences out of all predicted presences, and similarly for absences. Whilst the values of sensitivity and specificity were reasonable and values of negative predictive power were high in all models, the values of positive predictive power were low, i.e. the models over-predicted presences. Environmental conditions in terms of the variables we measured may be ideal in these areas where the predicted probability of occurrence is high yet birds are not observed. It is likely that populations of birds present on Alderney are relatively small in comparison to the potential area of suitable habitat available with limitations on suitable nesting sites or other factors on shore being more limiting than habitat at sea. Baldessarini et al [65] illustrate how positive predictive power significantly decreases and negative predictive power significantly increases as prevalence of occurrence decreases. Therefore a low positive predictive power may not necessarily signify a bad model. Additionally, birds in areas which are rarely used may not have been observed during the 16 surveys conducted in 2014. In addition, within the areas identified by the model as having a higher probability of occurrence, other factors such as competition [18] or local enhancement [17] may determine which of these areas are actually used. Habitual behaviour may also be an important factor, but not much is yet known about this. The relative importance of false positives and false negatives is highly dependent on the application of the predictions [41]. In the context of this study it is arguably less serious to over-predict presences than absences, as this would provide a precautionary approach to guide offshore developments. Our findings support previous suggestions that equal weightings of errors of omission (falsely predicted negative values) and commission (falsely predicted positive values) may not be a representative way to assess model accuracy [63]. Methods exist to define costs to false positives and false negatives, and weight these accordingly but these can be subjective and vary depending on the application [41].

#### Extending predictions into unobserved areas

Predictions in those sites in close proximity to the initial observation sites, where environmental conditions were similar, appeared to be superior to those further away, and certainty of predictions will decrease significantly in areas which were not previously surveyed. Ecological and oceanographic features can change at scales of only a few metres [20], and new areas may be subject to untested environmental or anthropogenic pressures. As models will never take into account all of the underlying variables explaining the distribution of seabirds, any predictions made outside of the study area should be interpreted cautiously.

### Recommendations

The near-shore, fine-scale distribution of seabirds in Alderney’s coastal waters is driven partially by depth, distance to the nearest seabird nest, distance to the intertidal zone and substrate type. Overall, the models performed reasonably well at identifying areas with suitable habitat types for all three groups, although other factors are undoubtedly involved in determining the near-shore fine-scale distributions of Alderney’s seabirds. In the absence of observation data, and as a precautionary approach, these models of habitat use could therefore be applied when recommending areas in which to limit human disturbance, for example in this instance boating and fisheries disturbance around Alderney could be directed away from rocky deep water areas near nests and intertidal zones. In this instance we could not view the site currently proposed for development of tidal turbines in Alderney (2km offshore) though this would not necessarily always be the case. Furthermore, installations may affect birds in close proximity to the site during construction and decommissioning and due to changes in energy and prey distribution as a result of mixing and sediment transport. Furthermore these changes in sedimentation processes which may occur through altered current regimes, and changes in tidal ranges due to the removal of energy around installations may effect near-shore seabird distributions. European shags may be particularly vulnerable in this respect due to their association with both the substrate type and proximity to intertidal zones [66].

Our example from Alderney shows that vantage point analyses are complementary to GPS tracking and ship-based and aircraft surveys in their ability to collect large quantities of highly accurate near-shore data at minimal expense. Additionally this method is site specific rather than colony specific allowing all birds in the area of interest to be monitored. However the observable distance from the shore is limited and detection rates become a problem at distances greater than approximately 1km. Additionally, observations cannot be conducted in poor weather conditions. Thus we suggest vantage-point observations are an ideal method in which to monitor bird distributions in near-shore coastal waters under the right conditions [67]. Therefore we suggest that when assessing potential impacts of marine disturbance on seabirds, observations and subsequent modelling to evaluate the active use of a site by seabirds can make a valuable contribution to the decision making process.

## Supporting Information

S1 Fig The environmental variables in the model; a) depth, b)substrate type, c) distance to the intertidal zone, d) distance to land. (DOCX)

S1 Table The number of scans conducted at each vantage point over the different states of tide and months of the year. (DOCX)
