# Supplementary material for: Predictive Modelling to Identify Near-Shore, Fine-Scale Seabird Distributions during the Breeding Season
Source: PLoS One. 2016 Mar 31;11(3):e0150592. doi: 10.1371/journal.pone.0150592 (PMC4816348; doi:10.1371/journal.pone.0150592)
Supplement: S1 Fig — (DOCX) [file pone.0150592.s001.docx]

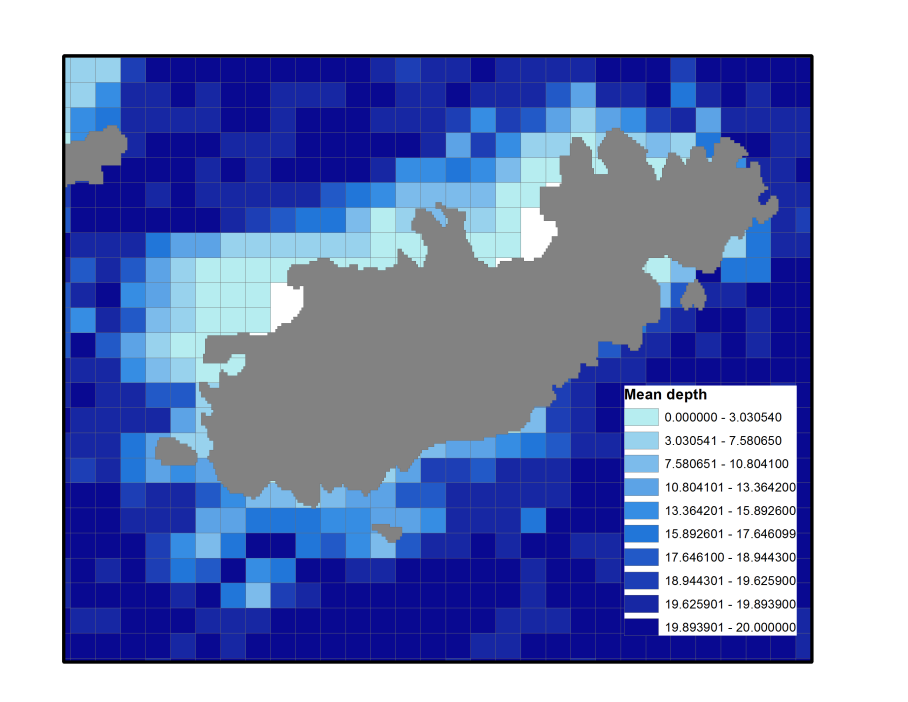

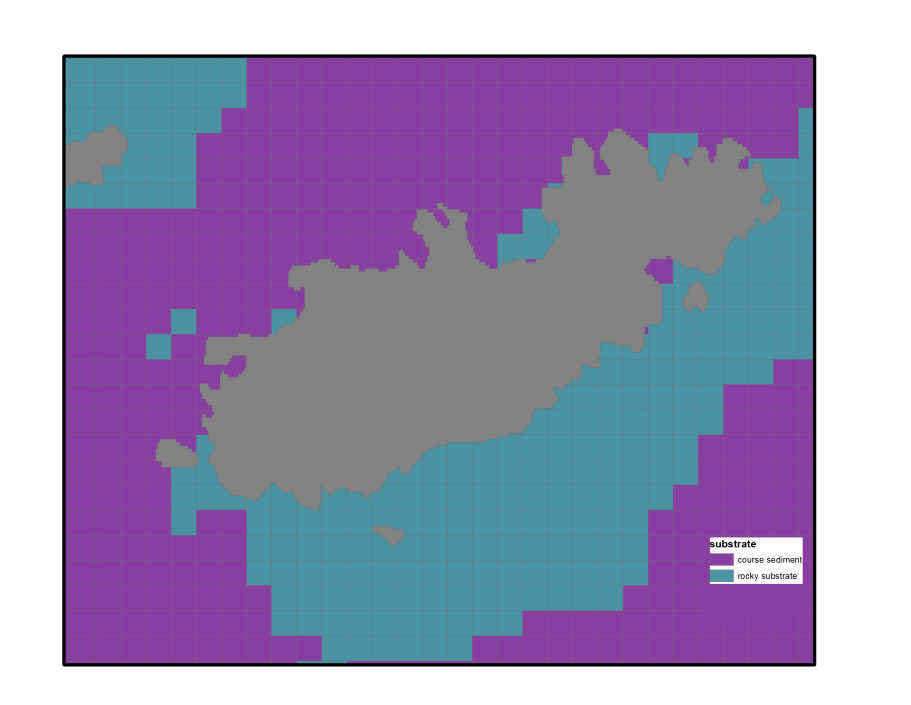

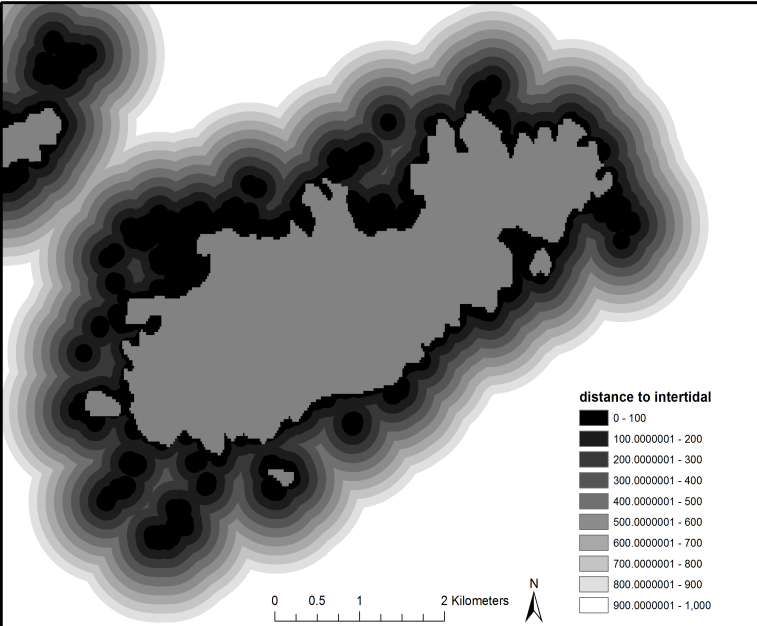

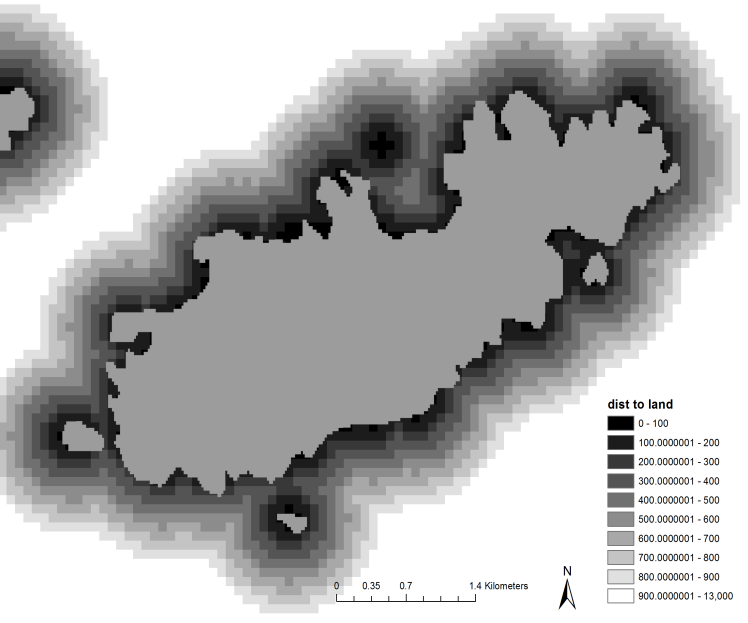


S1 Figure: The environmental variables in the model; a) depth, b)substrate type, c) distance to the intertidal zone, d) distance to land.
