# Supplementary material for: Predictive Modelling to Identify Near-Shore, Fine-Scale Seabird Distributions during the Breeding Season
Source: PLoS One. 2016 Mar 31;11(3):e0150592. doi: 10.1371/journal.pone.0150592 (PMC4816348; doi:10.1371/journal.pone.0150592)
Supplement: S1 Table — (DOCX) [file pone.0150592.s002.docx]

S1 Table. The number of scans conducted at each vantage point in order to measure bird distribution in Alderney’s near-shore water over the different states of tide and months of the year. The state of tide was not calculated for 2014 because it was not significant in the model based on 2013 data.

| Year | Month | Tide | Number of scans |
| --- | --- | --- | --- |
| 2013 | April | Ebb | 4 |
|  |  | Flood | 9 |
|  |  | Slack | 3 |
|  | May | Ebb | 6 |
|  |  | Flood | 11 |
|  |  | Slack | 4 |
|  | June | Ebb | 4 |
|  |  | Flood | 8 |
|  |  | Slack | 0 |
|  | July | Ebb | 1 |
|  |  | Flood | 10 |
|  |  | Slack | 6 |
| 2014 | April |  | 3 |
|  | May |  | 4 |
|  | June |  | 4 |
|  | July |  | 5 |
